# Prevalence and risk factors of upper gastrointestinal symptoms in community pharmacies in Spain: a cross-sectional study

**DOI:** 10.3389/fphar.2023.1162370

**Published:** 2023-06-13

**Authors:** María Puig-Moltó, Blanca Lumbreras, Elsa López-Pintor

**Affiliations:** ^1^ Department of Public Health, History of Science and Gynecology, Miguel Hernandez University, Elche, Spain; ^2^ CIBER of Epidemiology and Public Health, Centro de Investigación Biomédica en Red, Madrid, Spain; ^3^ Department of Engineering, Area of Pharmacy and Pharmaceutical Technology, Miguel Hernandez University, Elche, Spain

**Keywords:** community pharmacy, gastroesophageal reflux, functional dyspepsia, epigastric symptoms, retrosternal symptoms

## Abstract

**Background:** Frequently, the community pharmacies are the only points of consultation for upper-gastrointestinal symptomology. However, the heterogeneity of symptoms often limits the correct management of the patient. The study aim is to describe the epidemiological and clinical characteristics of patients with upper-gastrointestinal symptoms who ask for advice in community pharmacies.

**Methods:** A cross-sectional study was performed in 134 Spanish pharmacies (June-October 2022) and we included 1,360 patients. We collected sociodemographic, clinical variables and current medication data. The pharmacist evaluated the gastrointestinal symptoms through the application of the GERD Impact Scale (GIS questionnaire). Patients were classified into three groups according to their symptoms: epigastric, retrosternal and overlapping symptoms.

**Results:** Median age was 49 years (interquartile range 36–62 years) and 59.3% were women. Most patients reported overlapping symptoms (738%, 54.3%), 433 (31.8%) retrosternal and 189 (13.9%) epigastric symptoms. Patients with overlapping symptoms were more likely to associated consumption of foods and/or drinks and symptoms and showed lower scores on the GIS scale (median 26, IQR 20-30) than those with epigastric (median 32, IQR 29-33) and retrosternal (median 32, IQR 28-34) symptoms (*p* < 0.001). Patients in treatment with a combination of alginates and antiacids were more likely to think that it better alleviated their symptoms in all the patients included (*p* = 0.012).

**Conclusion:** More than half of the patients showed overlapping symptoms and were more likely to associate their symptoms with dietary habits and having poorer scores in the GIS scale. Clinical awareness of such overlapping condition would help optimize the management of patients with upper gastrointestinal symptoms in practice.

## 1 Introduction

Functional Dyspepsia (FD) and Gastroesophageal Reflux (GER) are the two most prevalent upper gastrointestinal disorders (Ford et al., 2015; Eusebi et al., 2018b). FD is defined by the Rome IV criteria (Drossman and Hasler, 2016) as the presence of symptoms thought to originate in the gastroduodenal region. FD includes patients with postprandial distress syndrome (PDS), characterized by meal-induced dyspeptic symptoms, and patients with epi gastric pain syndrome (EPS), which refers to epigastric pain or burning that does not occur exclusively postprandially, may occur during fasting, and may even improve with meal intake; however, both syndromes can coexist (de Bortoli et al., 2018). It is reported to affect approximately 20% of the general population (Tack et al., 2006). GER, defined by the presence of frequent heartburn or acid regurgitation, also affects 20% of the population (Locke et al., 1997). Both diseases are frequently chronic, often need treatment and affect patients’ quality of life (Ronkainen et al., 2006). In addition, GER can lead to Gastroesophageal Reflux Disease (GERD), a more severe and long-lasting condition.

Previous studies demonstrated an overlap between GER and FD, mainly in those patients with EPS. A previous population study in 3,831 subjects found that FD and GER occurred together more commonly than expected by chance (Guillemot et al., 2005; Tack et al., 2005; Ghoshal et al., 2011; Choung et al., 2012). Other studies have also shown this overlap in a considerable proportion of patients. A recent systematic review of studies including general population found that FD and GER overlap in more than 25% of individuals (Eusebi et al., 2018a). The possible causes could include the presence of confounding factors as the infection by *Helicobacter pylori* or shared pathophysiological mechanisms. In general, chronic gastritis, a known consequence of a chronic *H. pylori* infection, is known to lead to a reduction in gastric acid secretion and a reduced prevalence of GERD. Lower rates of gastritis were also observed in patients with FD (Quigley and Lacy, 2013). Previous evidence found that a common pathophysiological mechanism might underlie both conditions (Choung et al., 2012); nevertheless, few studies have examined pathogenetic mechanisms among patients in whom these disorders coexist or overlap. A previous systematic review summarized pathophysiological mechanisms that could contribute to this overlap, including esophageal acid exposure, gastric dysmotility and visceral hypersensitivity (Geeraerts et al., 2020). Another study supporting the hypothesis of an underlying pathophysiological mechanism in FD and GERD demonstrated that impaired gastric accommodation, a well-accepted pathophysiological mechanism in FD, was related to the presence of transient lower esophageal sphincter relaxations, which are the main mechanism underlying reflux episodes in both healthy subjects and patients with GERD (Pauwels et al., 2014). In addition, another important feature of FD is the presence of duodenal eosinophilia, which has recently been shown to be associated with an increased risk of new-onset GERD in FD (Ronkainen et al., 2019). The diagnosis of this condition is therefore unclear and the development of uniform definitions for the diagnosis of overlapping GERD and FD would help clinicians in the establishment of treatment approaches rather than symptom-based treatment.

For many patients the community pharmacies are the first points of consultation for gastrointestinal symptomologies, due to their accessibility (Tytgat et al., 2008). Moreover, mild symptoms are often treated with over-the-counter(OTC) medication and patients do not usually visit their general practitioner unless their symptoms worsen, partly due to the burden on primary care. The literature shows that pharmacists play an important role in the management of mild gastrointestinal symptoms, recognizing alarm symptoms, confirming the diagnosis, referring to a specialist when necessary, and guiding patients with OTC treatment (Boardman and Heeley, 2015). Although dispensing OTC are not the best option for clinical care, it is a common strategy in community pharmacies. OTC and proton pump inhibitors (PPi) treatments without prescription are also included in some clinical guidelines, due to their use in daily practice, as patients’ symptoms are sometimes intermittent and mild (Haag et al., 2009). A previous consensus-based guideline on the OTC management of gastroesophageal reflux disease with PPIs, reflected that apart from the physician referral, when necessary, the pharmacist could specify an OTC treatment to control symptoms (Holtmann et al., 2011). Furthermore, a previous Delphi consensus reached by an international group of experts, showed that the use of OTC and PPi was not associated with a significant risk in the treatment of symptoms under the supervision of healthcare professionals (Johnson et al., 2017).

Given that these disorders frequently coexist, the ability of the pharmacist to distinguish between FD, GER and FD-GER overlap, which affect patients’ health in different ways and imply different treatment approaches (Eusebi et al., 2018a), is essential for patient management. Although lifestyle interventions are required in patients with upper-gastrointestinal symptoms are treated with a proton-pump inhibitor (PPI) because many healthcare professionals assume that all upper gastrointestinal symptoms are due to acid reflux. However, patients with FD show a low response rate to this treatment and thus, their symptoms persist (Pinto-Sanchez et al., 2017). To establish an accurate diagnosis in the community pharmacy, the evaluation of relevant clinical and sociodemographic variables that distinguish patients with overlap symptoms from those who have either FD or GER could support these health professionals to better optimize treatment approaches in these patients.

Considering this scenario, a protocol to manage patients with upper-gastrointestinal symptoms has been developed by community pharmacists, general practitioners and gastroenterologists with the support of the Spanish Society of Community Pharmacy (SEFAC) and the Spanish Society of General Practitioners (SEMERGEN) (Semergen, 2019). The objectives of this protocol are a) to describe the epidemiological characteristics of patients with upper gastrointestinal symptoms who attend a community pharmacy, and b) to provide a precise protocol for the pharmacist in the management of patients with upper-gastrointestinal symptoms.

In this study we aimed to describe the different epidemiological and clinical characteristics of patients with upper gastrointestinal symptoms, who attend community pharmacies for advice or to obtain an OTC medication.

## 2 Materials and methods

### 2.1 Study design

A cross-sectional study was conducted to describe the sociodemographic and clinical characteristics of patients with upper gastrointestinal symptoms who attended community pharmacies between June and October 2022. This study is a part of a pre-post interventional study, whose protocol has already been published, which was carried out to evaluate the impact of a Professional Pharmaceutical Service on both patients’ symptoms and quality of life (López-Pintor et al., 2022).

### 2.2 Setting

We invited community pharmacists throughout the national territory to participate. In Spain, pharmacies are private healthcare establishments which work in concordance with the public health system and are subject to the health planning established by the Autonomous Communities. Although there is only one pharmacist owner per pharmacy, other pharmacists can work under the title of assistant pharmacist. Both pharmacists have the qualification requirements established by law, and are registered in the Official College of Pharmacists, according to Law 29/2006, the 26th July (BOE-A-2006-13554, 2006). In Spain, the Royal Decree 1/2015, the 24th July, regulates the sale of non-prescription medicines in pharmacies: non-prescription medicines can only be sold if they have been approved by the Spanish Agency for Medicines and Health Products.The pharmacist is obliged to inform patients about the correct use of medicines, precautions and side effects (BOE-A-2006-13554, 2006; BOE-A-2015-8343, 2015). At the end of 2021, there were 78,128 registered pharmacists and 22,198 community pharmacies in Spain. (Number of pharmacies in Spain 2007-2021, 2022).

### 2.3 Study population

We included patients ≥18 years who attended a community pharmacy due to the presence of upper gastrointestinal symptoms or who asked for treatment without prescription (over the counter -OTC- medication) for the treatment of these symptoms. We excluded those who asked for treatment for another person, or women with high-risk pregnancies. The study participants were only included if they had previously signed the informed consent form.

### 2.4 Sample size calculation

The sample size was estimated to fulfil the second of the objectives of the established protocol, which was to evaluate the impact of a Professional Pharmaceutical Service on both patients’ symptoms and quality of life. We estimated the number of people to assess 0.1 units of change over the GERD Impact Scale questionnaire (GIS), a 4-point Likert scale between 2 visits (from 2.2 to 1.1) with an SD of 0.6 through the estimation of the mean in repeated measures (López-Pintor et al., 2022). Taking into account an alpha risk of 0.05, two-sided test and a beta risk of 0.20 with a dropout of 20% we initially included 707 patients. However, we then increased the sample size to 1,200 patients (15 patients per variable) because we wanted to analyse different factors associated with relevant sociodemographic and clinical variables. This sample size allowed us to adequately describe the sociodemographic and clinical characteristics in patients with upper gastrointestinal symptoms who visited community pharmacies, which was the aim of the present study.

### 2.5 Recruitment procedure

The Spanish Society for Community Pharmacy (SEFAC), RECKITT and the Universidad Miguel Hernandez (UMH) asked a random sample of community pharmacists to voluntarily participate in our study, and we adjusted the sample size to the population of each autonomous community, until the required sample size was reached. We invited 411 community pharmacies to participate, but finally 134 (32.6%) community pharmacies from 23 Spanish provinces were included in the study. We estimated that each community pharmacy would have to include a minimum of 5 patients to achieve sample size. All registered pharmacists received prior training by the research team on the objective, methodology and procedures of the study. This training was accredited by the Miguel Hernandez University of Elche, where the research team is located.

### 2.6 Data collection

Patients who met the criteria were identified by a community pharmacist and then invited to participate in the study. First, the community pharmacist explained the study and gave them an information sheet (regarding privacy and details of the study) and if they agreed to participate, they signed the informed consent form. Patients were classified with an identification code: CA-III-PN (CA: autonomous community code; III: researchers initials; PN, and N represents participant’s number).

The pharmacist collected the sociodemographic and clinical information orally by means of a questionnaire. The sociodemographic characteristics included: a) age, b) sex (male or female), c) educational level: no studies, primary education, secondary education and university education. The clinical data included: a) body mass index (BMI) (kg/m2); b) reason for attending the pharmacy: seeking treatment advice or requesting OTC medication; c) requested treatments: antiacid monotherapy, a combination of alginates and antiacids, PPI; d) frequency of physical activity: every day, once or twice a week, 3-5 times a month and never; e) smoking habit: smokers, ex-smoker, never-smoker; f) frequency (daily/3-4 times a week, 1-4 times month, once a year or never) of consumption of foods and drinks associated with studied symptoms in the literature: alcoholic beverages, coffee, chocolate, tea, tomato, carbonated beverages, citrus food, spicy food, heavy or high-fat meals, and if the patients associate symptoms with their consumption of these items; g) onset of symptomatology (1–2 days ago, 3–4 days ago, 5–6 days ago, 7 days ago or more); h) presence of alarm criteria: symptoms (asthenia, dysphagia, recurrent vomiting, unexplained weight loss, gastrointestinal bleeding, severe pain, dyspnea, shortness of breath, nocturnal cough); intake of medicines often associated with symptoms (anti-inflammatory analgesics, bisphosphonates alendronates, calcium antagonists and/or nitrates, progesterone/oral contraceptives, tricyclic antidepressants/amitriptyline, theophylline, iron supplements, benzodiazepines, alpha-adrenergic antagonists/doxazosin and systemic corticosteroids), and diagnosis of gastrointestinal disorders or diseases (GERD, gastric ulcer, hiatus hernia, infection with *H. pylori*, food intolerances, colon disorders, gastritis); j) previous use of medicines to relieve symptoms, prescribed and/or OTC; k) GERD Impact Scale questionnaire (GIS) consisting in 5 questions: 1) location of symptoms, 2) effect of symptoms on eating and drinking habits, 3) effect of symptoms on sleep quality, 4) effect of symptoms on productivity at work and/or daily activities, 5) frequency of intake of unprescribed treatments. This questionnaire is validated in Spain (Nuevo et al., 2009).

### 2.7 Data analysis plan

We designed a database structured in accordance with the study variables. After the pharmacist had uploaded the information to the database, it was checked and validated by an external monitor. Only the principal researcher and the monitor had access to the database. The evaluation was carried out using IBM SPSS Statistics for Windows, version 27.0. Armonk, NY, USA: IBM Corp.

Patients were classified in 3 groups: a) patients with epigastric symptoms b) patients with retrosternal symptoms, and c) patients with overlapping epigastric and retrosternal symptoms. This classification was based on the one to five questions of GIS questionnaire related to localization of symptoms. Patients with epigastric symptoms were those who had a punctuation from 1 to 3 (frequency of symptoms: daily, often, or sometimes) in the question which refers to symptoms in the upper part of the stomach associated to the epigastric area and a 4 (never) in questions which refer to symptoms associated with the retrosternal area. Patients with retrosternal symptoms were those who had a punctuation from 1 to 3 (frequency of symptoms: daily, often, or sometimes) in questions which refer to symptoms associated with the retrosternal area and a punctuation of 4 (never) in the question which refers to symptoms associated to the epigastric area. Patients with overlapping symptoms were those who fulfilled both definitions.

Data were analysed and presented in descriptive form using absolute and relative frequencies with the 95% confidence interval, the GIS questionnaire responses were measured using a four-point Likert scale, and then a score is calculated, giving a number between 1 and 4. *p*-value <0.05 is considered significant.

A multivariate analysis was performed to measure the association between covariates (requested treatments, associations between alcoholic/carbonated drinks, spicy food and symptoms, frequency of avoiding eating or drinking foods because of the symptoms, use of prescribed/non prescribed treatment before to relief symptoms, previous treatment used) and retrosternal and both retrosternal and epigastric symptoms using a logistic regression model. We reported raw and multivariable adjusted odds ratios (ORs) with 95% CI and 0.05 < *p*-value was considered significant.

## 3 Results

### 3.1 Sociodemographic characteristics of the patients included in the study

**FIGURE 1:**
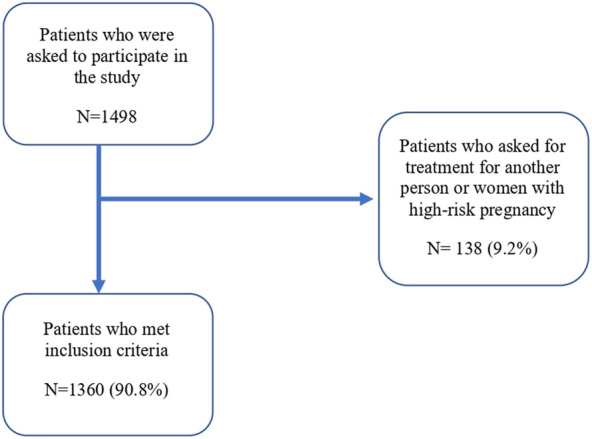
Flowchart of the participants of the study sample.

Of the 1,498 patients who attended a community pharmacy due to the presence of upper gastrointestinal symptoms or who asked for an OTC- medication, we included 1,360 (90.8%) patients (Figure 1). The included patients were divided into 3 groups according to their symptoms: 738 (54.3%) reported overlapping symptoms, 433 (31.8%) retrosternal symptoms, and 189 (13.9%) epigastric symptoms. Participants were predominantly female (807%, 59.3%), median (IQR) age was 49 years (36-62) and median (IQR) BMI was 25.8 kg/m2 (23.2–28.9). There were no differences between the different 3 groups according to age or sex. Most of the participants who went to the pharmacy were asking for an OTC medication to relieve their symptoms (773%, 57%). Most participants had university (474%, 34.9%) and secondary education (470%, 34.6%). According to lifestyle habits, 310 (22.8%) patients never practised physical activity and 699 (51.4%) were non-smokers (Table 1).

**TABLE 1:** Patients’ sociodemographic characteristics and reasons for attending a community pharmacy.

Variables N (%)	Total 1360 (100)	Epigastric symptoms 189 (13.9)	Retrosternal symptoms 433 (31.8)	Overlapping symptoms 738 (54.3)	*p*-value
**Sex**					*0.903*
- Female	807 (59.3)	111 (58.7)	254 (58.7)	442 (59.9)	
- Male	553 (40.7)	78 (41.3)	179 (41.3)	296 (40.1)	
**Age (years) (median, interquartile range)**	49 (36-62)	48 (36-60)	51 (38-64)	49 (36-61)	*0.345*
**BMI (kg/m** ^**2**^ **) (median, interquartile range)**	25.8 (23.1–28.9)	25.2 (22.6–28.4)	25.8 (23.3–28.6)	25.9 (23.1–29.0)	*0.488*
**Reason for consultation**					*0.277*
Seeking treatment advice for the symptoms	533 (39.2)	78 (41.3)	158 (36.5)	297 (40.3)	
Requesting over-the counter medication	774 (57)	108 (57.1)	258 (59.6)	408 (55.4)	
Both	52 (3.8)	3 (1.6)	17 (3.9)	32 (4.3)	
**Requested treatments**					*0.001*
- Antiacid monotherapy	573 (70.1)	98 (89.1)	191 (70.5)	284 (65.1)	
- Alginates in combination with antiacids	108 (13.2)	4 (3.6)	32 (11.8)	72 (16.5)	
- Proton pump inhibitors	82 (10.0)	6 (5.5)	28 (10.3)	48 (11.0)	
**Educational level**					*0.096*
- No studies	63 (4.6)	8 (4.2)	21 (4.8)	34 (4.6)	
- Primary education	321 (23.6)	56 (29.6)	83 (19.2)	182 (24.7)	
- Secondary education	470 (34.6)	57 (30.2)	173 (40.0)	240 (32.5)	
- University education	474 (34.9)	65 (34.4)	146 (33.7)	263 (35.6)	
- NS/NC	32 (2.4)	3 (1.6)	10 (2.3)	19 (2.6)	
**Frequency of physical activity**					*0.839*
- Every day	381 (28.0)	57 (30.2)	125 (28.9)	199 (27.0)	
- Once or twice/a week	415 (30.5)	60 (31.7)	129 (29.8)	226 (30.6)	
- 3-5 times/a month	254 (18.7)	28 (14.8)	83 (19.2)	143 (19.4)	
- Never	310 (22.8)	44 (23.3)	96 (22.2)	170 (23.0)	
**Smoking habits**					*0.215*
- Smokers	316 (23.2)	55 (29.1)	88 (20.3)	173 (23.1)	
- Ex-smoker	345 (25.4)	44 (23.3)	116 (26.7)	185 (25.1)	
- Non-smokers	699 (51.4)	90 (47.6)	229 (52.9)	380 (51.5)	

There were statistical differences according to the treatment requested. Antacid monotherapy was the most requested treatment in all groups (70.1%) and mainly in patients with epigastric symptoms (89.1%) in comparison with patients with retrosternal symptoms (70.5%) and overlapping symptoms (65.1%). A combination of alginate and antiacid (16.5%) and PPI (11%) were more frequently requested in patients with overlapping symptoms (11%) than in patients with epigastric (5.5%) and retrosternal symptoms (10.3%) (*p* = 0.001).

### 3.2 Dietary habits of the patients included in the study

Table 2 shows the patients’ dietary habits according to consumption frequency. Coffee (957%, 70.4%), tomato (1,018%, 74.9%) and citrus fruits (700%, 51.5%) were more frequently consumed (daily/3-4 times per week). In contrast, alcohol beverages (551%, 40.5%), tea (883%, 64.9%), carbonated beverages (647%, 47.6%) and spicy food (868%, 63.8%) were less frequently consumed (once a year/never). There were no significant differences between the frequency of consumption and the different patients’ gastrointestinal symptoms.

**TABLE 2:** Frequency of food/drink consumption according to the patients’ symptoms.

Variables N (%)	Total	Epigastric symptoms 189 (13.9)	Retrosternal symptoms 433 (31.8)	Overlapping symptoms 738 (54.3)	*p*-Value
**Alcoholic beverages**					*0.507*
- Daily/3-4 times a week	378 (27.8)	47 (24.9)	119 (27.5)	212 (28.7)	
- 1-4 times a month	431 (31.7)	58 (30.7)	148 (34.2)	225 (30.5)	
- Once a year/never	551 (40.5)	84 (44.4)	166 (38.3)	301 (40.8)	
**Coffee**					*0.309*
- Daily/3-4 times a week	957 (70.4)	127 (67.2)	311 (71.8)	519 (70.3)	
- 1-4 times a month	67 (4.9)	7 (3.7)	26 (6.0)	34 (4.6)	
- Once a year/never	336 (24.7)	55 (29.1)	96 (22.2)	185 (25.1)	
**Chocolate**					*0.582*
- Daily/3-4 times a week	532 (39.1)	70 (37.0)	165 (38.1)	297 (40.2)	
- 1-4 times a month	448 (32.9)	58 (30.7)	144 (33.3)	246 (33.3)	
- Once a year/never	380 (27.9)	61 (32.3)	124 (28.6)	195 (26.4)	
**Tea**					*0.356*
- Daily/3-4 times a week	288 (21.2)	46 (24.3)	78 (18.0)	164 (22.2)	
- 1-4 times a month	189 (13.9)	25 (13.2)	65 (15.0)	99 (13.4)	
- Once a year/never	883 (64.9)	118 (62.4)	290 (67.0)	475 (64.4)	
**Tomato**					*0.155*
- Daily/3-4 times a week	1018 (74.9)	150 (79.4)	316 (73.0)	552 (74.8)	
- 1-4 times a month	213 (15.7)	23 (12.2)	81 (18.7)	109 (14.8)	
- Once a year/never	129 (9.5)	16 (8.5)	36 (8.3)	77 (10.4)	
**Carbonated beverages**					*0.701*
- Daily/3-4 times a week	369 (27.1)	45 (23.8)	113 (26.1)	211 (28.6)	
- 1-4 times a month	344 (25.3)	51 (27.0)	109 (25.2)	184 (24.9)	
- Once a year/never	647 (47.6)	93 (49.2)	211 (48.7)	343 (46.5)	
**Citrus fruits**					*0.437*
- Daily/3-4 times a week	700 (51.5)	98 (51.9)	213 (49.2)	389 (52.7)	
- 1-4 times a month	360 (26.5)	56 (29.6)	121 (27.9)	183 (24.8)	
- Once a year/never	300 (22.1)	35 (18.5)	99 (22.9)	166 (22.5)	
**Spicy food**					*0.992*
- Daily/3-4 times a week	158 (11.6)	22(11.6)	49(11.3)	87(11.8)	
- 1-4 times a month	334 (24.6)	44(23.3)	107(24.7)	183(24.8)	
- Once a year/never	868 (63.8)	123(65.1)	277(64.0)	468(63.4)	
**Heavy or high-fat meals**					*0.554*
- Daily/3-4 times a week	268 (19.7)	38 (20.1)	80 (18.5)	150 (20.3)	
- 1-4 times a month	595 (43.8)	82 (43.4)	181 (41.8)	332 (45.0)	
- Once a year/never	497 (36.5)	69 (36.5)	172 (39.7)	256 (34.7)	

There were differences between patients’ gastrointestinal symptoms and the food that patients associated with the symptoms. Patients who associated symptoms with consumption of alcohol beverages (*p* < 0.001), chocolate (*p* < 0.001), tomato (*p* < 0.001), carbonated beverages (*p* < 0.001), citrus fruits (*p* < 0.001), coffee (*p* = 0.017), tea (*p* = 0.014) and spicy food (*p* = 0.001) were more likely to present overlapping symptoms (Table 3).

**TABLE 3:** Associations between consumption of foods/drinks and patients´ symptoms.

Variables N (%)	Total	Epigastric symptoms 189 (13.9)	Retrosternal symptoms 433 (31.8)	Overlapping symptoms 738 (54.3)	*p*-value
- **Alcoholic beverages**	361 (26.5)	36 (19.0)	96 (22.2)	229 (31.0)	*<0.001*
- **Coffee**	349 (25.7)	50 (26.5)	90 (20.8)	209 (28.3)	*0.017*
- **Chocolate**	295 (21.7)	32 (16.9)	70 (16.2)	193 (26.2)	*<0.001*
- **Tea**	60 (4.4)	9 (4.8)	9 (2.1)	42 (5.7)	*0.014*
- **Tomato**	455 (33.5)	60 (31.7)	109 (25.2)	286 (38.8)	*<0.001*
- **Carbonated beverages**	263 (19.3)	23 (12.2)	63 (14.5)	177 (24.0)	*<0.001*
- **Citrus fruits**	392 (28.8)	50 (26.5)	82 (18.9)	260 (35.2)	*<0.001*
- **Spicy food**	471 (34.6)	52 (27.5)	130 (30.0)	289 (39.2)	*0.001*
- **Heavy or high-fat meals**	779 (57.3)	104 (55.0)	233 (53.8)	442 (59.9)	*0.101*

### 3.3 Patients’ clinical characteristics according to the GIS score

Onset of symptoms was 1–2 days for most of patients (607%, 44.6%), and 7 days or more (556%, 40.9%). Patients whose symptoms had started 1–2 days ago were more likely to have only retrosternal symptoms (236%, 54.5%) than only epigastric (96%, 50.8%) or overlapping symptoms (275%, 35.3%) (*p* < 0.001). Patients whose symptoms had started 7 days or more ago were more likely to have overlapping symptoms (311%, 42.1%) than either only retrosternal (143, 33%) or only epigastric symptoms (60%, 31.7%) (*p* < 0.001) (data not shown).

Median GIS score was 21 (IQR 26-34) (Table 4). The group of patients with overlapping symptoms (median 26, IQR 20-30) had worst punctuation in the GIS score than patients with only epigastric (median 32, IQR 29-33) or retrosternal symptoms (median 32, IQR 28-34) (*p* < 0.001). Patients with overlapping symptoms (51%, 6.9%) reported daily difficult to sleep because of the symptoms more frequently than those with epigastric (9%, 4.8%) and retrosternal symptoms (14%, 3.2%). Moreover, patients with overlapping symptoms (68%, 9.2%) reported daily difficult to eating or drinking because of the symptoms more frequently than those with epigastric (24%, 5.5%) and retrosternal symptoms (3%, 1.6%). Daily difficult to being fully productive in their job and daily activities because of the symptoms was more frequently reported in patients with overlapping symptoms (28%, 3.8%) than those with epigastric (2%, 1.1%) and retrosternal symptoms (5%, 1.2%).

**TABLE 4:** GIS score according to the patients’ symptoms.

Variables N (%)	Total	Epigastric symptoms 189 (13.9)	Retrosternal symptoms 433 (31.8)	Overlapping symptoms 738 (54.3)	*p*-Value
1.How often have you had the following symptoms					
**a. Pain in your chest or behind your breastbone?**					*<0.001*
- Daily	46(3.4)	0	14(3.2)	32(4.3)	
- Often	158(11.6)	0	37(8.5)	121(16.4)	
- Sometimes	172(12.7)	0	43(9.9)	129(17.5)	
- Never	983(72.3)	189(100)	339(78.3)	455(61.7)	
**b. Burning sensation in your chest or behind the breastbone**					*<0.001*
- Daily	115(8.5)	0	32(7.4)	83(11.3)	
- Often	298(21.9)	0	80(18.5)	218(29.6)	
- Sometimes	291(21.4)	0	101(23.3)	190(25.8)	
- Never	655(48.2)	189(100)	220(50.8)	246(33.4)	
**c. Regurgitation or acid taste in your mouth?**					*<0.001*
- Daily	112(8.2)	0	25(5.8)	87(11.8)	
- Often	326(24)	0	81(18.7)	245(33.2)	
- Sometimes	360(26.5)	0	122(28.2)	238(32.2)	
- Never	561(41.3)	189(100)	205(47.3)	167(22.7)	
**d. Pain or burning in your upper stomach?**					*<0.001*
- Daily	161(11.8)	40(21.2)	0	121(16.4)	
- Often	425(31.3)	76(40.2)	0	349(47.4)	
- Sometimes	340(25)	73(38.6)	0	267(36.2)	
- Never	433(31.9)	0	433(100)	0	
**e. Sore throat or hoarseness that is related to your heartburn or acid reflux?**					*<0.001*
- Daily	52(3.8)	0	11(2.5)	41(5.6)	
- Often	161(11.8)	0	39(9)	122(16.6)	
- Sometimes	154(11.3)	0	50(11.5)	104(14.1)	
- Never	992(73)	189(100)	333(76.9)	470(63.8)	
**2.How often have you had difficulty getting a good night´s sleep because of your symptoms?**					*<0.001*
- Daily	74 (5.4)	9 (4.8)	14 (3.2)	51 (6.9)	
- Often	274 (20.1)	35 (18.5)	54 (12.5)	185 (25.1)	
- Sometimes	412 (30.3)	52 (27.5)	110 (25.4)	250 (33.9)	
- Never	600 (44.1)	93 (49.2)	255 (58.9)	252 (34.1)	
**3.How often have your symptoms prevented you from eating or drinking any of the foods you like?**					*<0.001*
- Daily	95(7.0)	3(1.6)	24(5.5)	68(9.2)	
- Often	238(17.5)	29(15.3)	39(9.0)	170(23.0)	
- Sometimes	290(21.3)	49(25.9)	70(16.2)	171(23.2)	
- Never	737(54.2)	108(57.1)	300(69.3)	329(44.6)	
**4.How frequently have your symptoms kept you from being fully productive in your job or daily activities?**					*<0.001*
- Daily	35(2.6)	2(1.1)	5(1.2)	28(3.8)	
- Often	105(7.7)	8(4.2)	16(3.7)	81(11.0)	
- Sometimes	122(9.0)	17(9.0)	17(3.9)	88(11.9)	
- Never	1098(80.7)	162(85.7)	395(91.2)	541(73.3)	
**5.How often do you take additional medication other than what the physician told you to take?**					*<0.001*
- Daily	93(6.8)	8(4.2)	22(5.1)	63(8.5)	
- Often	114(8.4)	21(11.1)	24(5.5)	69(9.3)	
- Sometimes	205(15.1)	27(14.3)	51(11.8)	127(17.2)	
- Never	948(69.7)	133(70.4)	336(77.6)	479(64.9)	
**GIS score (median, interquartile range)**	31 (26-34)	32 (29-33)	32 (28-34)	26 (20-30)	*<0.001*

*GIS: gastroesophageal reflux disease impact scale.

Women were more likely to report daily burning sensation in her chest or behind the breastbone than men (83%, 10.3% vs. 32%, 5.8%, *p* = 0.007). Women (88%, 10.9%) also reported more frequently presence of regurgitation or acid taste in her mouth daily than men (88%, 10.9% vs. 24%, 4.3%, *p* < 0.001), and more frequently sore throat or hoarseness that is related to her heartburn or acid reflux daily than men (41%, 5.1% vs. 11%, 2%, *p* = 0.030). In addition, women reported daily difficulty to sleep more frequently than men (78%, 9.7% vs. 17%, 3.1%, *p* < 0.001) and difficulties in daily activities (29%, 3.6% vs. 6%, 1.1%, *p* < 0.001). However, there were no differences in the median GIS score between women and men.

### 3.4 Alarm criteria detected in the patients included in the study

Of the 1,390 patients included in the study, 190 (14%) had asthenia and 118 (8.7%) severe pain, 258 (19%) patients had taken anti-inflammatory analgesics and 140 (10.3%) benzodiazepines, 171 (12.6%) patients had a diagnosis of hiatus hernia and 83 (6.1%) a diagnosis of GERD.

There were differences between the different alarm criteria detected between patients with overlapping symptoms and those with epigastric and retrosternal symptoms: Asthenia (136%, 18.4%%; 13, 6.9%, and 41%, 9.5%, respectively, *p* < 0.001), dysphagia (53%, 7.2%; 2%, 1.1% and 24%, 5.5%, respectively, *p* = 0.006), recurrent vomiting (58%, 7.9%; 1%, 0.5%% and 13%, 3%, respectively, *p* < 0.001), dyspnoea (73%, 9.9%; 1%, 0.5% and 20%, 4.6%, respectively, <p< .001) severe pain (85%, 11.5%; 10%, 5.3% and 23%, 5.3%, respectively, *p* < 0.001), intake of tricyclic antidepressants/amitriptyline (20%, 2.7%; 0 and 3%, 0.7%, respectively, *p* = 0.005), diagnosis of GERD (55, 7.5%; 3, 1.6% and 25, 5.8%, respectively, *p* = 0,001) and gastric ulcer (31%, 4.2%; 7, 3.7%, and 6%, 1.4%, respectively, *p* = 0.029) were more likely to be detected in patients with overlapping symptoms than in patients with epigastric or retrosternal symptoms.

### 3.5 Previous treatments

Most patients (883%, 64.9%) had taken treatment to relief their symptoms previously to the inclusion in the study. Patients with epigastric symptoms were more likely to have an O-T-C medication (91%, 79.8%) than patients with retrosternal symptoms (158%, 60.3%) and those with overlapping symptoms (299%, 59%) (*p* = 0.006) (Table 5). Treatment with PPI (268%, 36.3%) were more frequently used in patients with overlapping symptoms than in patients with epigastric (42%, 22.2%) and retrosternal symptoms (128%, 29.6%). Patients with epigastric and retrosternal symptoms in treatment with a combination of alginate and antiacid were more likely to think that it better alleviated their symptoms (17%, 100% and 57%, 98.3% respectively) than patients with overlapping symptoms (100%, 86.2%), *p* = 0.012.

**TABLE 5:** Description of type of treatment used previously attending the pharmacy and treatment outcome in relieving symptoms.

Variables N(%)	Total	Epigastric symptoms 189(13.9)	Retrosternal symptoms 433 (31.8)	Overlapping symptoms 738 (54.3)	*p*-Value
**Did you use any treatment before visiting the pharmacy?**	883 (64.9)	114(60.3)	262 (60.5)	507 (68.7)	*0.006*
**Type of treatment used**					*<0.001*
- Prescribed	252 (28.5)	23 (20.2)	77 (29.4)	151 (30)	
- Non prescribed	548 (62.1)	91 (79.8)	158 (60.3)	299 (59)	
- Both	83 (9.4)	0	27 (10.3)	56 (11)	
**Previous treatment used**					
1) Alginates in combination with antiacids	191 (14.0)	17(9.0)	58(13.4)	116 (15.7)	*0.053*
*How did it go?*					*0.012*
- *Well*	*174 (91.1)*	*17 (100)*	*57 (98.3)*	*100 (86.2)*	
- *Average-bad*	*17 (8.9)*	*0*	*1 (1.7)*	*16 (13.8)*	
2) Antiacid monotherapy	351(25.80)	60(31.7)	101(23.3)	190(25.7)	*0.087*
*How did it go?*					*0.494*
- Well	313 (89.2)	55 (91.7)	92 (91.1)	166 (87.4)	
- *Average-bad*	38 (10.8)	5 (8.3)	9 (8.9)	24 (12.6)	
3) Proton pump inhibitor	438(32.2)	42(22.2)	128(29.6)	268(36.3)	*<0.001*
*How did it go?*					*0.315*
- Well	361 (82.4)	34 (81)	111 (86.7)	216 (80.6)	
- *Average-bad*	77 (17.6)	8 (19)	17 (13.3)	52 (19.4)	

In multivariate analysis comparing patients with overlapping symptoms with either retrosternal or epigastric symptoms, patients who associated alcoholic beverages with symptoms were more likely to have overlapping symptoms (aOR 1.41, 95% CI 1.01–1.85) than patients with either epigastric or retrosternal (*p* = 0.016). Patients who associated citrus with symptoms were more likely to have overlapping symptoms (aOR 1.40, 95% CI 1.05–1.86) than patients with either epigastric or retrosternal (*p* = 0.020). Patients with overlapping symptoms were less likely to never have difficulty to sleep than those with either epigastric or retrosternal symptoms (aOR 0.53, 95% CI 0,30-0.95 (*p* = 0.032). Patients with overlapping symptoms were less likely to never have difficulty to being fully productive in their job or daily activities than those with either epigastric or retrosternal symptoms (aOR 0.34, 95% CI 0,14-0.87 (*p* = 0.024).

## 4 Discussion

In this study, 54.3% of patients presented overlapping symptoms, 31.8% of patients presented retrosternal symptoms and 13.9% of patients, epigastric symptoms. There were no differences in the clinical and sociodemographic variables associated with patients with different symptoms. However, patients with overlapping symptoms were more likely to relate their symptoms to their dietary habits and showed alarm criteria more frequently than those with epigastric and retrosternal symptoms. In addition, patients with overlapping symptoms had poorer scores in the GIS scale and were more likely to have taken a previous treatment with PPI. Antacid monotherapy was the most frequently requested treatment by all patient groups and most of the patients had previously used an OTC medication to relieve their symptoms. Patients with a previous treatment based on a combination of antacid and alginate were more likely to acknowledge an improvement in their symptoms.

The prevalence of overlapping symptoms in patients with upper-gastrointestinal symptoms in patients attending a community pharmacy was higher than previous frequencies shown in general population. A previous systematic review examined the overlapping symptomatology of GER and FD in the general population, and they found that both conditions overlap in more than 25% of individuals (Eusebi et al., 2018a). However, the included studies showed a high heterogeneity (ranging from 3% to 51%) due to the different population characteristics and the various instruments used to evaluate the symptoms.

Our results support previous evidence that FD-GER overlap is a separate clinical entity since patients with this condition were more likely to have more severe symptoms and other differential characteristics than those with either only epigastric or only retrosternal symptoms. A community study in Belgium undertook a survey of FD-GER symptoms in a random sample of 2,025 subjects (Piessevaux et al., 2009). As in our study, they found that patients with overlapping symptoms were more likely to have more severe and higher frequency of dyspeptic symptoms compared to dyspeptic subjects without heartburn.

Our results showed no significant association between lifestyle variables such as dietary habits or smoking habit and the three clinical conditions. Nevertheless, there were significant results when the patients themselves associated their symptomatology with specific dietary habits. Patients with overlapping symptoms more frequently associated their symptoms with the consumption of alcoholic beverages and citrus fruits than patients with either only epigastric or only retrosternal symptoms. In a previous systematic review, citrus fruits, carbonate beverages, spicy and fried foods were found to increase the risk of developing GER (Heidarzadeh-Esfahani et al., 2021). However, the authors concluded that more studies are needed to clarify the specific effect of diet on the disease. Although other systematic reviews stated that weight loss, smoking habit and intake of alcohol, chocolate, fatty food, spicy food and citrus showed a relationship with developing GER, they also concluded that additional clinical studies were required (Kaltenbach et al., 2006; Festi et al., 2009).Changing dietary habits are the first-line recommendation in gastrointestinal disorders, so carrying out further studies to evaluate specific food groups that can produce these symptoms could be important to improve or avoid symptomatology.

As in previous studies, we assessed impairment of quality of life using the GIS scale. We found similar scores in patients with either only retrosternal or only epigastric symptoms, but patients with overlapping symptoms showed the lowest scores. Some studies that used SF-36 found a greater impairment of quality of life in patients with overlapping symptoms which was consistent with our results (Kaji et al., 2010; Lee et al., 2014). In addition, patients with overlapping symptoms were more likely to show worse scores in the items related with insomnia, and impact of the symptoms on their eating/drinking intakes and on their daily activities than patients with either only epigastric or only retrosternal symptoms. Self-reported insomnia has been found to be a risk factor for FD-GER overlap compared to either only FD or only GER in several studies (Schey et al., 2007; Gerson and Fass, 2009), which showed that this association might be directional.

Most of the patients included in the study requested a specific treatment to relieve their symptoms than to seek pharmaceutical advice. In a Spanish study, 13% of patients had taken medication to control gastroesophageal reflux symptoms and antacid monotherapy was the most used medication (Ponce et al., 2006). In the present study, similarly, antiacid monotherapy was the most requested treatment followed by PPi therapy. PPi treatment is the first line of treatment included in the clinical guidelines of GER symptoms, and in a previously mentioned study, PPi treatment was more frequently used in patients with overlapping upper gastrointestinal symptoms in comparison to those with either only esophagic or only retrosternal symptoms (Choung et al., 2012). The presence of epigastric symptoms, however, has been showed as a risk factor for poorer response to PPi in GER patients (Matsuhashi et al., 2015). Therefore, overlapping FD and GER might have important effects on therapeutic responses. The combination of alginate and antiacid was the treatment with the highest level of patient satisfaction regarding symptom relief, especially in patients with either only epigastric or only retrosternal symptoms, but also showed positive results among those with overlapping symptoms. In the literature, some studies showed that alginates were more effective than placebo or antiacid monotherapy and had a similar effect to PPIs in the management of GER symptoms (Leiman et al., 2017; Zhao et al., 2020). In a German study, patients on chronic PPi treatment with additional alginates treatment showed an improvement in their GERD-Q score (Müller et al., 2019).

As many patients in our study went to a pharmacy requesting treatment, it is essential to review patients’ previous treatment considering their symptomatology to offer optimum symptom management and to optimize treatment approaches.

### 4.1 Study limitations

This study has several limitations. First, categorizing patients might be a limitation due to the heterogeneity of symptoms, but we included a pre-training of the research pharmacists and a monitor to validate the data collected to reduce potential bias. Another limitation is the fact that given that we performed a cross-sectional study, we were unable to establish a temporal relationship between the symptoms and clinical and sociodemographic variables.

One of the strengths is that it is the first study describing clinical and sociodemographic characteristics of patients with upper gastrointestinal symptoms attending a community pharmacy. Given the sample size and the distribution of the pharmacies along the national territory, we think our results could be generalised to other settings. In addition, we categorized patients into three groups of symptoms (epigastric, retrosternal and overlapping symptoms) to identify the different symptoms and variables associated with more precision, thus reflecting clinical practice. In addition, the present study was carried out following the outline of a protocol developed by gastroenterologists, primary care providers and community pharmacists (López-Pintor et al., 2022).

## 5 Conclusion

In conclusion, patients with overlapping symptoms were the most frequent clinical condition detected in the patients included in our study. In addition, these patients were more likely to associate their symptoms with dietary habits, had poorer scores in the GIS scale and used more PPi therapy before visiting the pharmacy than those with either only epigastric or only retrosternal symptoms. These findings suggest that overlap patients may represent a different condition and thus need different management strategies in the community pharmacy.

## Data Availability

The raw data supporting the conclusion of this article will be made available by the authors, without undue reservation.

## Abbreviations

FD, functional dyspepsia; GERD, gastroesophageal reflux disease; GER, gastroesophageal reflux; GERD impact scale questionnaire (GIS questionnaire); OTC, over the counter medication.
